# A suspected case of hyponatraemia induced rhabdomyolysis: a case report

**DOI:** 10.1186/s12882-022-02787-7

**Published:** 2022-05-11

**Authors:** Edward Reakes, Douglas Drak, David Gracey

**Affiliations:** 1 Renal Unit, Royal Prince Alfred Hospital, Camperdown, NSW 2050 Australia; 2 Wagga Wagga Base Hospital, Wagga Wagga, NSW 2650 Australia; 3 Central Clinical School, University of Sydney, Camperdown, NSW 2050 Australia

**Keywords:** Hyponatraemia, Rhabdomyolysis, Acute kidney injury

## Abstract

**Background:**

Hyponatraemia is a documented but under-recognised cause of rhabdomyolysis, with the contrasting treatment strategies for the two conditions posing a unique challenge. Balancing the need for aggressive fluid replacement for the treatment of rhabdomyolysis, with the risk of rapidly correcting hyponatraemia is imperative.

**Case presentation:**

A 52-year-old gentleman with a background of HIV infection and hypertension presented with seizures following methamphetamine use, acute water intoxication, and thiazide use. He was found to have severe hyponatraemia, and following initial correction with hypertonic saline, was commenced on a fluid restriction. After two days he developed abdominal wall and thigh pain, along with oliguria. Laboratory data demonstrated markedly elevated creatine kinase levels and deteriorating renal function. A diagnosis of rhabdomyolysis and severe acute kidney injury was made and aggressive fluid replacement commenced, leading to full resolution of the hyponatraemia, rhabdomyolysis and acute kidney injury.

**Conclusion:**

Hyponatraemia-induced rhabdomyolysis is rare but can cause significant morbidity and mortality if left untreated. Physicians should consider measuring creatine kinase levels in all patients presenting with severe hyponatraemia, particularly in the presence of other risk factors for rhabdomyolysis. Fluid replacement strategies must be considered in relation to the relative onset and risk of over-correcting hyponatraemia.

## Background

Hyponatraemia is a documented but under-recognised cause of rhabdomyolysis [1–9]. The interaction of the two conditions poses a significant challenge to clinicians due to the conflicting nature of their managements. Vigorous hydration remains imperative in the treatment of rhabdomyolysis, however in the presence of hyponatraemia, the danger of rapidly correcting the sodium level cannot be overlooked. We present the case of a 52-year-old patient with HIV who developed hyponatraemia and subsequent rhabdomyolysis, in the context of other contributing factors including methamphetamine abuse, combined anti-retroviral therapy, and seizure activity.

## Case presentation

A 52-year-old gentleman with a three year history of well controlled HIV infection, long-standing hypertension and ongoing methamphetamine and alcohol misuse inhaled approximately 0.1 g of methamphetamine and developed neck discomfort. Attributing this to hypertension, he consumed 6 L water over the course of thirty minutes and self-administered three tablets of his regular antihypertensive Avapro HCT (Irbesartan 300/ Hydrochlorothiazide 12.5 mg). Within an hour, the patient had a tonic-clonic seizure lasting one to two minutes. A second seizure after the arrival of the ambulance service was terminated with IV midazolam.

**Fig. 1:**
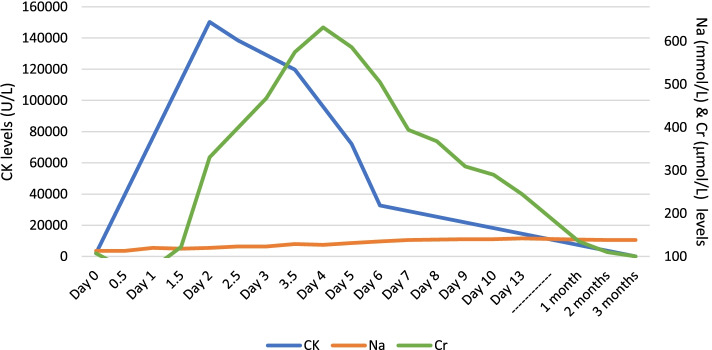
Serum creatine kinase (CK), sodium (Na) and creatinine (Cr) levels over the course of hospital admission and follow up

On arrival to the emergency department, the patient’s Glasgow coma score (GCS) was 14, he was afebrile with observations in normal limits. The patient was post ictal with no evidence of head injury, clinically euvolemic, and cardiorespiratory and abdominal examinations were normal. The initial laboratory work revealed severe hyponatraemia (sodium 113 mmol/L), hypochloraemia (chloride 73 mmol/L), elevated creatine kinase (CK 1979 U/L), lactic acidosis (pH 7.1, bicarbonate 7 mmol/L, lactate 17 mmol/L), with preserved kidney function (serum creatinine 107 μmol/L, eGFR 68 mL/min/1.73m^2^) (Table 1, and Fig. 1). Serum osmolality was 251 mmol/kg, with a urine sodium and urine osmolarity of 129 mmol/L and 474 mmol/kg, respectively. The patient had no known history of previous electrolyte disturbance or kidney injury. A non-contrast CT scan of the brain revealed no intracranial bleeding. Given the severe hyponatraemia and presence of neurological sequelae, 150 mL hypertonic saline 3% was commenced. The patient also received IV 1500 mg Keppra. He was transferred to the intensive care unit (ICU) for electrolyte monitoring and commenced on a fluid restriction of 700 mL, aiming to correct the serum sodium level no more than 6–10 mmol in 24 h.

**Table 1:** Serum and urine biochemistry chemistry results

	Day 0	Day 1	Day 2	Day 3	Day 4	Day 5	Day 6	Day 7	Day 8
Serum Creatinine (μmol/L)	107	122	399	522	632		394	368	309
Serum Sodium (mmol/L)	113	119	123	126	127		138	139	140
Serum Creatinine Kinase (U/L)			150,324	119,790		72,034			
Serum Osmolality (mOsm/L)	251		273						
Urine Osmolality (mOsm/L)	474		305						
Urine Sodium (mmol/L)	129		14

After two days the patient developed abdominal wall and upper thigh pain, with associated weakness. The patient also developed dark tea-coloured urine and oliguria. Further laboratory work demonstrated an elevated CK 150,324 U/L, creatinine 330 mmol/L, Na 120 mmol/L, K 4.6 mmol/L, phosphate 2.09 mmol/L, ALT 276 U/L, AST 1229 U/L, with a normal pH on blood gas. Repeat urine sodium was 14 mmol/L, osmolality 305 mmol/kg and a CT KUB demonstrated peri-nephric stranding. He remained clinically euvolemic.

The patient was diagnosed with severe acute kidney injury (AKI) secondary to rhabdomyolysis and fluid restriction was ceased. Aggressive fluid therapy was initiated with a 1 L stat bolus of normal saline 0.9% and further maintenance normal saline 0.9% at a rate of 125 ml/hr. The creatinine peaked on Day 4 of admission (632 μmol/L) and hyperkalaemia (5.8 mmol/L) was treated with calcium resonium on Day 6 of admission, by which point he had a positive fluid balance of approximately 5 L since commencement of intravenous fluids. The patient did not require renal replacement therapy. Close monitoring of the electrolytes continued, and the patient was discharged after ten days of admission.

Six weeks later, the sodium had normalised, and the creatinine had improved to 135 mmol/L. There was no proteinuria or haematuria on urinalysis. His BP was 150/90 mmHg. The patient was commenced on amlodipine 10 mg daily, in replacement of Avapro HCT and encouraged to drink 2 L of fluid per day.

## Discussion

Hyponatraemia-induced rhabdomyolysis is reported in the literature, although limited to small case series and case reports [1–8]. It is predominantly seen in the psychiatric population, specifically patients with chronic schizophrenia and psychogenic polydipsia [1–5], although cases are also reported in association with adrenal insufficiency [6, 7] and a further case is described in a patient who compulsively drank excess water to treat a ureteric calculus [8]. This report describes a case whereby excess water intake was provoked by methamphetamine use, leading to severe hyponatraemia, and in the context of seizures and combined antiretroviral therapy, the subsequent development of rhabdomyolysis.

Although the exact mechanism underlying hyponatraemia-induced rhabdomyolysis is unknown, a number of hypotheses have been proposed [1, 2, 9]. Rapid shifts in osmotic pressures and consequent cell swelling may trigger localised increases in cytosolic calcium [9]. The increase in intracellular calcium activates calcium dependent proteases leading to cell lysis of the myocytes [9]. In our case, the acute water intoxication event precipitated rapid onset of hyponatraemia, cerebral oedema characterised by seizures, and thus this hypothesis seems plausible.

Hyponatraemia may also provoke rhabdomyolysis by reducing the efflux calcium via the sodium-calcium exchanger in myocyte walls due to the reduced transmembrane gradient [9]. The increased intracellular calcium activates proteases and lipases leading to myocyte lysis [9]. Despite these proposed hypotheses however, a causal association between hyponatraemia and rhabdomyolysis remains to be demonstrated. Replication of hyponatraemia-induced rhabdomyolysis has been unsuccessful in an animal model [10].

Hypertonic saline 3% was used to treat the severe hyponatraemia due to the development of seizures, improving the serum sodium level to 120 mmol/L. As the initial urinary sodium level was 129 mmol/L, syndrome of inappropriate anti-diuretic hormone was diagnosed and fluid restriction was commenced. This posed a unique challenge on Day 2 of admission, as contrasting aggressive fluid replacement was required for the treatment of rhabdomyolysis and developing severe AKI. Rhabdomyolysis results in a ‘functional’ dehydration due to capillary damage and intravascular volume depletion and thus aggressive rehydration improves renal perfusion and eliminates nephrotoxic substrates [11]. Overzealous correction of hyponatraemia and the risk of osmotic demyelination syndrome had to be considered, as rapid shifts in plasma osmolality can reverse established cerebral oedema quickly, leading to central and pontine demyelination [12].

Hypertonic saline is indicated when neurological complications ensue with hyponatraemia, as in our case, and careful monitoring in a high dependency setting with repeated serum sodium levels is required. However, whilst this may prevent further neurological complications and remove the substrate for further rhabdomyolysis, it does not allow for the aggressive fluid replacement required to prevent renal injury. To overcome the opposing needs of the two conditions’ treatment strategies, continuous renal replacement therapy (CRRT) has been demonstrated to be effective in a previous case report [9]. CRRT was initiated preferentially to intermittent haemodialysis, in a patient with severe hyponatraemia (104 mmol/L) secondary to psychogenic polydipsia and chronic schizophrenia, presenting with acute anuric renal failure. Secombe et al. diluted a commercially available dialysate to reduce the sodium concentration, allowing for slower correction of the established hyponatraemia [9]. They slowly increased the concentration of sodium in the replacement fluid as the hyponatraemia improved, until the serum sodium was >125 mmol/L, before switching to continuous veno-venous haemodiafiltration to optimise the patient’s biochemical profile and remove excess water.

In contrast, our patient did not develop any indications for renal replacement therapy, and as the onset of hyponatraemia was acute (< 48 h), aggressive fluid replacement was initiated. Due to the acute nature of the onset of hyponatraemia, the risk of osmotic demyelination syndrome was substantially less [12]. Managing the AKI and risk of acute renal failure therefore took precedent. The decision was effective, with the sodium normalising slowly over the following 72 h, without any neurological complications.

It is important to note that a number of other coexisting causes of rhabdomyolysis may have contributed to this case. The patient had used methamphetamine, an established cause of rhabdomyolysis secondary to vasoconstriction induced muscle ischaemia and prolonged muscle contraction-induced ATP depletion [13–15]. The contribution of methamphetamines is likely limited, however, given the small dose that was administered, to which the patient was tolerant of higher doses, without having previously developed rhabdomyolysis. Although the patient presented with seizures, another recognised cause of rhabdomyolysis [16], their contribution however was similarly unlikely to have been significant. Elevation in CK level is proportional to the type and frequency of seizure activity and when considered with our case, the CK levels observed were far higher than expected for the seizure activity alone [16]. With less than 2% of patients presenting with seizures developing CK elevations of greater than 50 times the upper limit of normal [17], the modest seizure activity witnessed is unlikely to have contributed significantly to the ensuing rhabdomyolysis.

Several previous case reports have implicated combined anti-retroviral therapy (cART) with the onset of rhabdomyolysis [18–24]. The cART regimen used to treat HIV infection in our patient consisted of dolutegravir, an integrase inhibitor, and emtricitabine (FTC) and tenofovir alafenamide (TAF), nucleoside and nucleotide reverse transcriptase inhibitors (NRTIs). Several case reports describe rhabdomyolysis in association with integrase inhibitors, with four reported in association with raltegravir use [19–22] and another with dolutegravir use as part of the combination dolutegravir-abacavir-lamivudine (Triumeq) [23]. The use of cART may therefore have contributed to the development of rhabdomyolysis in this case. While further precipitants may have contributed to the rhabdomyolysis, we believe methamphetamine use, seizures and ART to be the most likely.

Rhabdomyolysis is a rare but significant complication of hyponatraemia with associated renal morbidity. This case illustrates the importance of recognising the association of the two conditions and should encourage physicians to consider regularly measuring CK levels in the presence of severe hyponatraemia. The challenge posed by the conflicting treatment strategies involved must be carefully considered, with novel strategies including individualised CRRT used to mitigate the risk of rapidly correcting the serum sodium level in the presence of acute renal failure caused by rhabdomyolysis. Future studies to elucidate the underlying mechanism would be useful to further understand hyponatraemia-induced rhabdomyolysis, in order to guide treatment strategies.

## Data Availability

All data supporting the case is included in the manuscript.
